# Comparison between Culture and a Multiplex Quantitative Real-Time Polymerase Chain Reaction Assay Detecting *Ureaplasma urealyticum* and *U. parvum*

**DOI:** 10.1371/journal.pone.0102743

**Published:** 2014-07-21

**Authors:** Maria Frølund, Eva Björnelius, Peter Lidbrink, Peter Ahrens, Jørgen Skov Jensen

**Affiliations:** 1 Department of Microbiology and Infection control, Sexually Transmitted Infections, Research and Development, Statens Serum Institut, Copenhagen, Denmark; 2 Department of Dermatovenereology, Huddinge University Hospital, Karolinska Institutet, Huddinge, Sweden; The University of Melbourne, Australia

## Abstract

A novel multiplex quantitative real-time polymerase chain reaction (qPCR) for simultaneous detection of *U. urealyticum* and *U. parvum* was developed and compared with quantitative culture in Shepard's 10 C medium for ureaplasmas in urethral swabs from 129 men and 66 women, and cervical swabs from 61 women. Using culture as the gold standard, the sensitivity of the qPCR was 96% and 95% for female urethral and cervical swabs, respectively. In male urethral swabs the sensitivity was 89%. The corresponding specificities were 100%, 87% and 99%. The qPCR showed a linear increasing DNA copy number with increasing colour-changing units. Although slightly less sensitive than culture, this multiplex qPCR assay detecting *U. urealyticum* and *U. parvum* constitutes a simple and fast alternative to the traditional methods for identification of ureaplasmas and allows simultaneous species differentiation and quantitation in clinical samples. Furthermore, specimens overgrown by other bacteria using the culture method can be evaluated in the qPCR.

## Introduction

Originally recognised as tiny (T)-strain mycoplasmas due to their small colony size, ureaplasmas were first isolated from human genital samples in 1954 and assigned to a new genus and species, *Ureaplasma (U.) urealyticum*, in 1974 [1]. Based on differences in sensitivity to manganese, the polypeptide patterns, and percent identity by DNA-DNA hybridization experiments, *U. urealyticum* was first divided into two biovars [2] and, after further research, the two biovars were assigned species status. *U. urealyticum* (formerly biovar 2 or T980) comprise the serotypes 2, 4, 5, 7–13, and *U. parvum* (formerly biovar 1 or parvo) comprise serotypes 1, 3, 6 and 14 [3], [4]. In the following the term ureaplasmas will be used, where the species has not been determined, or where it is irrelevant.

Ureaplasmas are a part of the urethral and vaginal flora and can be isolated from the urogenital tract from up to 40% of asymptomatic individuals [5]–[8]. However, undifferentiated ureaplasmas have also been associated with acute [9] and chronic non-gonococcal urethritis (NGU) [10], preterm delivery [11], and complications in preterm infants [12]. While differences for the two ureaplasma species in perinatal morbidity and mortality has not been studied much, it has been suggested, that only *U. urealyticum* has pathogenic potential in NGU [6], [7], [13], [14], while other studies have failed to show this association [15], [16].

Molecular biology based techniques, such as PCR, can distinguish between the two species [7], [17]–[19], whereas standard culture identifies the bacteria to the genus level only. More recently, quantitative real-time PCR (qPCR) estimating the bacterial load in a sample has been developed [20], and quantitative detection might prove clinically important. We developed a multiplex TaqMan qPCR with an internal control for inhibition to detect, quantify, and discriminate *U. urealyticum* and *U. parvum* simultaneously. Although species-specific PCR assays have been previously used to differentiate the ureaplasma species in clinical specimens, this is, to our knowledge, the first published validation of a ureaplasma multiplex qPCR assay regarding sensitivity, specificity, and accuracy of quantification in clinical samples using quantitative culture as the gold-standard.

## Materials and Methods

### Ethics Statement

Patients were enrolled in the study irrespective of the reason for attending the clinic and provided oral consent after having received written and oral information regarding the study. The Regional Ethics Committee for Stockholm, Karolinska Institutet, Stockholm, Sweden approved the study, protocol no 32/95 with an amended protocol no 392/01. Written consent and registration of oral consent was not needed according to the approval of the Ethics Committee.

### Patients and Samples

Samples were collected from patients attending the outpatient STD clinic at Huddinge University Hospital, Sweden, between August 1997 and November 2001 as a part of a study of the clinical aspects of *M. genitalium* infections [21]. Patients were enrolled in the study irrespective of the reason for attending the clinic.

All samples had been tested for *Chlamydia trachomatis* and *Mycoplasma genitalium* by PCR as previously described [21]. In patients where gonorrhoea was suspected, *Neisseria gonorrhoeae* was detected by culture.

Swabs were collected from the urethra and cervix with an ear, nose and throat (ENT) cotton-tipped aluminium swab and placed in a tube with 1.8 ml of SP4 mycoplasma broth medium [22]. After the clinical examination, the patient collected 15 ml of first voided urine, of which a 4 ml sample was mailed to Copenhagen together with the swab specimen. DNA preparation from swabs and urine samples were performed at the day of receipt and then frozen at −20°C. Swab samples were frozen at −80°C, until ureaplasma culture was performed.

For this study, urethral swabs from 129 men and 66 women, together with 61 cervical swabs, were selected for ureaplasma culture and qPCR. The samples included in the present study were a subset of the samples included in a study of *M. genitalium* infections [21], but blinded to the patients' infection status or symptoms, as the present study was only designed for comparison of the qPCR assays with culture. The selected specimens were consecutively collected in the time periods May–August 2000 and March–May 2001 without any knowledge of infection status, clinical signs or symptoms. Of the female urethral and cervical swabs, 60 were pairs from the same patients.

### Culture for Ureaplasmas

Swabs were stored at −80°C for 1–31 months (median 13 months) before culture. The SP4 medium used for transport of the specimen was thawed, and 200 µl were transferred to 1.8 ml of 10 C medium with urea creating the first 10-fold dilution. From these samples, series of four additional 10-fold dilutions were made and the highest dilution that produced a colour change (yellow to pink) after incubation in closed vials under atmospheric conditions at 37°C was regarded as containing 1 colour-changing unit (CCU). Another 200 µl of the SP4 medium was inoculated into 1.8 ml of Shepard's 10 C medium without urea [23] creating a single 10-fold dilution. The medium without urea served as a back-up for recovery of the ureaplasma strains, in the event that growth in the urea containing medium was so rapid that the ureaplasmas died. Approximately 25 µl from the medium without urea, corresponding to 2.5 µl of the original SP4 medium, was transferred to U-agar [23] and incubated at 37°C in 5% CO_2_. Typical ureaplasma colonies were identified on agar to confirm that the colour-change was caused by ureaplasmas and not by other urease-producing bacteria.

### Polymerase Chain Reaction

#### Sample Preparation

Sample preparation for PCR from swabs was performed by adding 100 µl of the SP4 transport medium to 300 µl of a 20% Chelex-100 slurry (BioRad, Hercules, CA) in TE buffer (10 mM Tris-HCl [pH 8.0], 1 mM EDTA). The mixture was vortexed for 1 minute and incubated at 95°C for 10 minutes.

Urine samples were concentrated by centrifugation of 1,800 µl at 30,000×*g* for 15 min. The supernatant was aspirated carefully in order not to dislodge the pellet and yet to leave only a minimal amount of urine in the tube. Subsequently, 300 µl of the 20% Chelex-100 slurry was added and the tube was vortexed for 1 minute and incubated at 95°C for 10 minutes.

The DNA extracts were stored at −20°C, until PCR was performed. Samples were vortexed before PCR and centrifuged briefly. Five µl of the supernatant was used as template for PCR's corresponding to approximately 1.5 µl of the original swab sample and 37.5 µl of the original urine sample. Urine samples were used only for resolving discrepancies between culture and PCR.

#### Selection of Primers and Probes

A common forward primer U195F (5′GCAAGAAGACGTTTAGCTAGAGGTTT) and two overlapping species specific reverse primers U8R (5′CACGAGCAGATTGCATTAAGTCAG) and U3R (5′CGAGCAGATTGCATTAGGTCAG), respectively, were designed to amplify a 127-bp fragment of the urease gene in *U. urealyticum* serovar 2, 4, 5 and 7–13 and a 125-bp fragment of the urease gene in *U. parvum* serovar 1, 3, 6 and 14. Two overlapping species specific probes U8 (5′FAM-TAATTACTGACCACGTAGTGGA-*minor groove binder* (MGB)) and U3 (5′VIC-TTTAATTACTGATCATGTAATGGA-MGB) (Life Technologies, Paisly, UK) were designed to target the urease gene in *U. urealyticum* and *U. parvum*, respectively. The urease gene was chosen as target, because it is considered conserved within each of the ureaplasma species [24].

#### Construction of positive controls

Primers U138F (5′ATGAATCTATCATTAAGAGAAARTCC) and U1036R (5′AGAYTCTTCACCATAAGTAGTTAAGT) targeting both ureaplasma species were used to amplify an 899-bp fragment of the urease gene in *U. urealyticum^T^* (serotype VIII; ATCC 27618) and *U. parvum^T^* (serotype III; ATCC 27815) from genomic DNA.

The amplicons were gel purified and eluted in TE buffer. The DNA concentration was determined with the Qubit dsDNA Assay Kit (Life Technologies). Ten-fold dilutions containing 1–100,000 genome equivalents (geq) of *U. urealyticum* and *U. parvum* DNA per µl were made in TE buffer (pH 8.0) containing 1 µg/ml of calf thymus DNA (D-8661; Sigma-Aldrich, St. Louis, MO, USA) as carrier DNA.

#### Construction of an internal control for inhibition

To avoid false negative results caused by Taq DNA polymerase inhibitors or suboptimal PCR reaction conditions, an internal process control (IPC) was constructed as previously described [25]. Primers amplifying a part of the phage lambda genome were selected and synthesized with the urease gene primers U195F and U3R added to the 5′end of the lambda primers (U195F-IPC: GCAAGAAGACGTTTAGCTAGAGGTTT**CCGGGACGTATCATGCT** and U3R-IPC: CGAGCAGATTGCATTAGGTCAG**ACCGCTCAGGCATTTGCT** ). Sequences in bold face type correspond to the phage lambda sequence. PCR products containing the binding sites of the urease primers were obtained by amplification of 1 ng of purified phage lambda DNA. The 190-bp amplicon was gel purified, and a 10-fold dilution of the IPC was added to separate master mixes. The dilution of the IPC that had no influence on the threshold cycle (Ct) number for purified ureaplasma DNA at the limit of detection was used in this assay. The phage lambda sequence was detected with the IPC-R probe (5′TAMRA-TCCTTCGTGATATCGGACGTTGGCTG-BHQ-2) (TAG Copenhagen, Copenhagen, Denmark).

#### TaqMan Assay

The qPCR assay was performed in a final volume of 50 µl containing 1X PCR buffer (20 mM Tris-HCl (pH 8.4), 50 mM KCl), 125 µM each of dATP, dCTP, and dGTP, and 250 µM dUTP, 5 mM MgCl_2_, 10% glycerol, 1 µM each of U3R and U195F primer, and 2 µM U8R primer, 150 nM IPC-R TaqMan probe, 150 nM each of the U3 and U8 TaqMan MGB probes, 5 µl of the appropriate dilution of the IPC, 1 µl of 0.83 µM 6-carboxy-x-rhodamine (ROX) reference dye (a 30-fold dilution of 50X ROX, Life Technologies in PCR-grade water), and 2 U *Taq* DNA polymerase (Platinum Taq, Life Technologies).

An ABI 7500 real-time PCR instrument (Applied Biosystems) was used with a 96-well block, MicroAmp Optical 96-well reaction plates and thermal cycling conditions comprising 1 cycle step of 50°C for 1 s then 95°C for 10 min followed by 50 cycles of denaturation at 95°C for 15 s and annealing and extension at 60°C for 1 min.

Standard curves were generated by analyzing 10-fold dilutions of purified *U. urealyticum* and *U. parvum* DNA amplicons containing 1–100,000 geq per µl. All tests were performed in duplicate with 5 µl DNA template and the reported results represent the mean of the two quantities. All qPCR results are presented as DNA copies per µl in the original SP4 transport medium, except when compared to culture, where the qPCR results were converted to geq per 200 µl original SP4 medium, corresponding to the culture inoculum.

To determine the limit of detection of the multiplex TaqMan assay, purified *U. urealyticum* and *U. parvum* DNA corresponding to 250, 50, 25, 5 and 3 geq template of each species was tested in 10 replicates. Logistic regression analysis was used to determine the amount of DNA that could be detected with 95% likelihood.

### Specificity of the TaqMan Assay

In silico analyses of the primers and probes were carried out using BLAST and in vitro analyses of the specificity of the TaqMan assay was evaluated by testing *U. diversum* (ATCC 43321), *U. gallorale* (ATCC 43346) and *U. cati* (NCTC 11710). Furthermore, the assay was tested against other bacterial species harbouring the urease gene; *Enterobacter cloacae, Proteus vulgaris, Proteus mirabilis, Yersinia enterocolitica, Klebsiella pneumoniae, Klebsiella oxytoca, Morganella morganii, Streptococcus salivarius, Helicobacter pylori, Haemophilus influenzae*, *H. parainfluenzae* and *H. haemolyticus*. All strains were from the Department of Clinical Microbiology at Statens Serum Institut. DNA was released by boiling of a colony with Chelex as described for the clinical samples, and 5 µl of each lysate was used as template.

### Evaluation of Competition in the Multiplex Assay in Simulated Mixed Infection

To examine the ability to detect both ureaplasma species in a sample, mixtures with different known amounts of DNA of *U. urealyticum* and *U. parvum* (*U. urealyticum*:*U. parvum*) were prepared in ratios 1∶10,000, 1∶1,000, 1∶100, 1∶10 and 1∶1, and 10,000∶1, 1,000∶1, 100∶1 and 10∶1 with a total DNA concentration of approximately 10^5^ copies/µl.

### Methods for Resolving Discrepancies between qPCR and Culture

For discrepant culture and qPCR results, the original samples were recovered from −80°C after prolonged storage and tested again by culture and qPCR in the same way as in the initial testing and, in addition, with another conventional PCR assay targeting the 16S rRNA gene in *U. urealyticum* and *U. parvum* using the primer sets P6 and U8, and P6 and U3, respectively [17]. The U3 primer was modified by adding one base: 5′TAGAA**A**GTCGCTCTTTGTGG. The thermo cycling conditions of the 16S rRNA gene assay was modified: a single cycle of 95°C for 5 min. followed by 10 cycles each of 95°C for 15 s, 72°C for 15, and 72°C for 90 s with a 1°C touch-down reduction for each cycle in the annealing step, and thereafter 40 cycles each of 95°C for 15 s, 62°C for 15 s, and 72°C for 90 s. In addition, stored DNA extracts of the samples and the matching urine samples from 2000 and 2001 were also tested by both PCR assays.

When evaluating the discrepant samples, a patient was considered ureaplasma positive, when a positive culture result was obtained from either initial testing or re-testing, or when a patient was positive in both PCR assays in any sample sites and species determination agreed.

### Statistical Analysis

StatsDirect statistical software, version 2.7.8 (StatsDirect Ltd., Cheshire, UK) was used for all statistical analyses. Fishers Exact Test was used to compare proportions between groups, the Mann-Whitney test when comparing two groups with continuous variables and the Kruskal-Wallis test when comparing more than two groups with continuous variables. McNemar's test was used to compare proportions in urethral and cervical swabs sampled from the same women. P-values <0.05 (two-sided) were considered statistically significant throughout.

GraphPad Prism 5 (GraphPad Software, Inc. La Jolla, CA, USA) was used to illustrate the relation between organism load by qPCR and CCU.

## Results

### Limit of Detection

The standard curves generated by 10-fold dilutions of *U. urealyticum* and *U. parvum* DNA were linear over a range of 6 log units with an *r* ^2^-value of >0.99. The calculated limit of detection that would be positive for 95% of the reactions analysed was 2.9 and 2.7 geqs for *U. urealyticum* and *U. parvum*, respectively.

### Specificity of the TaqMan Assay

BLAST against Genbank showed that the primers and probes were specific. Testing of the specificity panel did not give rise to detectable amplification and no inhibition was observed by the multiplex TaqMan assay.

### Patient samples

**Figure 1:**
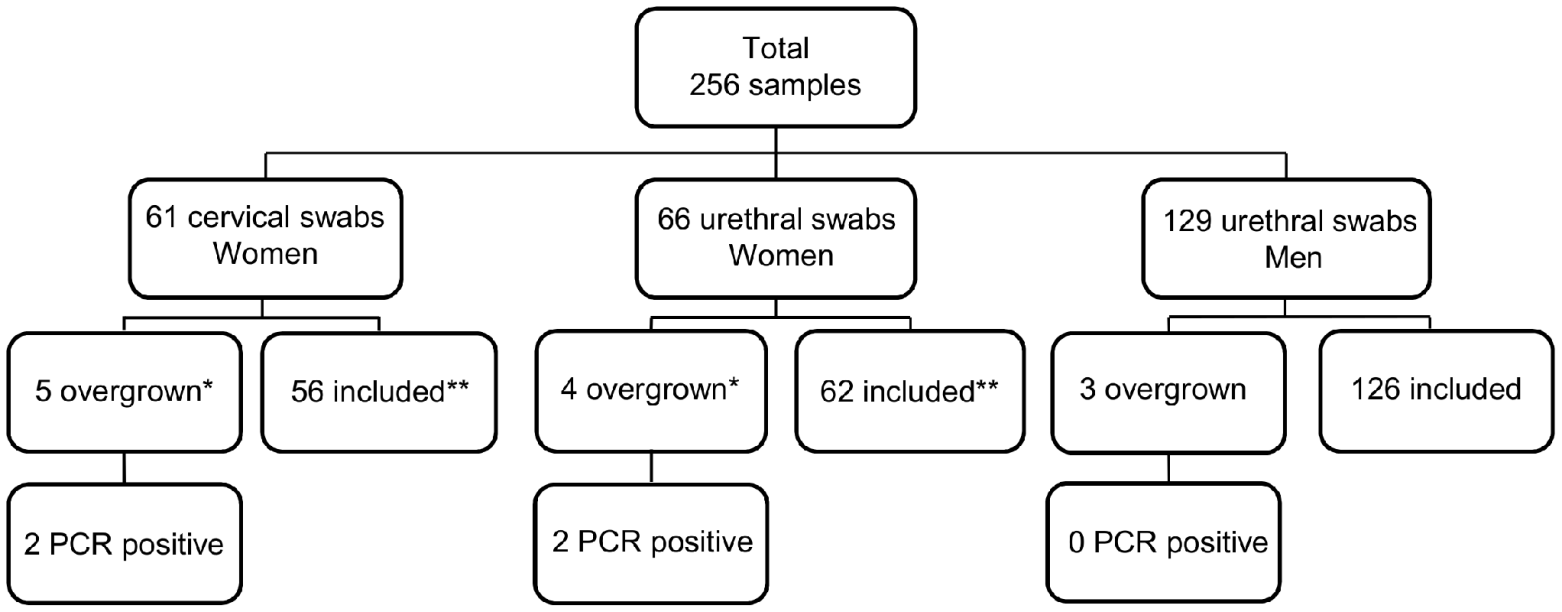
Flow-chart showing the distribution of samples included in the study. * Four had a matching urethral- or cervical swab sample from the same woman which was also overgrown. One excluded cervical swab sample was not overgrown in the matching urethral swab. ** Fifty-five had a paired urethral- or cervical swab sample from the same woman.

A total of 256 samples were examined; 129 male urethral swabs, and 66 urethral- and 61 cervical swabs from a total of 67 women. Three, four and five of these samples, respectively, were overgrown with other bacteria in culture leaving 126, 62 and 56, respectively, for comparison (Figure 1). Out of the 12 overgrown samples, four were qPCR positive; two were urethral samples and two cervical samples (pairs from two patients) (Figure1). One patient was *U. parvum* positive with 7.8×10^1^ copies/µl in both urethra and cervix, while the other was co-infected with both ureaplasma species and a total of 6.3×10^3^ copies/µl in the urethra and 2.9×10^3^ copies/µl in the cervix.

### Comparison between qPCR and Culture

Of the male urethral swabs, 31% (39 of 126) were positive by both methods, 0.8% (1 of 126) only by qPCR and 4% (5 of 126) only by culture (Table 1). Among the female urethral swabs 69% (43 of 62) were positive by both methods, none only by qPCR and 3% (2 of 62) only by culture (Table 2). Of the female cervical swabs 70% (39 of 56) were positive by both methods, 4% (2 of 56) only by qPCR and 4% (2 of 56) only by culture, respectively (Table 3). None of the samples inhibited the qPCR amplification.

**Table 1:** Detection of ureaplasmas in 126 male urethral specimens by culture and qPCR.

	No. of specimens			No. of culture-positive specimens in CCU group 10^1^–10^5^				
	Total	Culture positive	Culture negative	10^1^	10^2^	10^3^	10^4^	10^5^
qPCR positive	40	39	1	0	1	4	11	23
qPCR negative	86	5	81	0	3	0	0	2
Total	126	44	82	0	4	4	11	25
Diagnostic performance		89%	99%	-	25%	100%	100%	92%

**Table 2:** Detection of ureaplasmas in 62 female urethral specimens by culture and qPCR.

	No. of specimens			No. of culture-positive specimens in CCU group 101–105				
	Total	Culture positive	Culture negative	10^1^	10^2^	10^3^	10^4^	10^5^
qPCR positive	43	43	0	0	2	5	9	27
qPCR negative	19	2	17	0	0	1	1	0
Total	62	45	17	0	2	6	10	27
Diagnostic performance		96%	100%	-	100%	83%	90%	100%

**Table 3:** Detection of ureaplasmas in 56 female cervical specimens by culture and qPCR.

	No. of specimens			No. of culture-positive specimens in CCU group 10^1^–10^5^				
	Total	Culture positive	Culture negative	10^1^	10^2^	10^3^	10^4^	10^5^
qPCR positive	41	39	2	1	6	4	8	20
qPCR negative	15	2	13	1	0	1	0	0
Total	56	41	15	2	6	5	8	20
Diagnostic performance		95%	87%	50%	100%	80%	100%	100%

Out of the 60 paired female samples, four pairs and one cervical sample were overgrown by other bacteria. Of the remaining 55 paired samples 43 urethral samples were ureaplasma positive by culture (Table 4). Three (7%) of the 43 positive urethral samples were ureaplasma negative in the matching cervical sample, while none of the 40 paired cervix positive samples were urethra negative by culture (p>.99, McNemar's test) (Table 4). Comparing the distribution of ureaplasma positives in cervical and urethral samples by qPCR in the 60 paired female samples, 42 and 17 pairs were positive and negative in both sample sites, respectively, while one (2%) urethral swab was positive and the matching cervical swab negative (p = 0.25, McNemar's test) (Table 5).

**Table 4:** Distribution of ureaplasma positive and negative samples in 55 paired urethral and cervical swabs collected from the same woman as determined by culture.

		No. of cervical swabs		
		Culture positive	Culture negative	Total N
No. of urethral swabs	Culture positive	40	3	43
	Culture negative	0	12	12
Total N		40	15	55*

Five paired samples were not evaluable as they were overgrown with other bacteria.

*No difference in the distribution (p>0.99 McNemar's test).

**Table 5:** Distribution of ureaplasma positive and negative samples in 60 paired urethral and cervical swabs collected from the same woman as determined by qPCR.

		No. of cervical swabs		
		qPCR positive	qPCR negative	Total N
No. of urethral swabs	qPCR positive	42	1	43
	qPCR negative	0	17	17
Total N		42	18	60*

*No difference in the distribution (p = 0.25 McNemar's test).

Using culture results as reference, the sensitivity of the qPCR was 89% (39 of 44), [95% CI 63–100] in male urethral swabs, 96% (43 of 45), [95% CI 69–100] in female urethral swabs and 95% (39 of 41), [95% CI 68–100] in cervical swabs (Table 1, 2 and 3). These sensitivities were not significantly different (p = 0.47). Corresponding specificities were 99% (81 of 82), [95% CI 78–100], 100% (17 of 17), [95% CI 58–100], and 87% (13 of 15), [95% CI 46–100] (Table 1, 2 and 3). The difference in specificity, however, did not reach statistical significance (p = 0.06).

Only two of all 130 culture positive samples had a titer of 1–10 CCUs. Both were female cervical swabs, and one was positive by qPCR (*U. urealyticum*).

**Figure 2:**
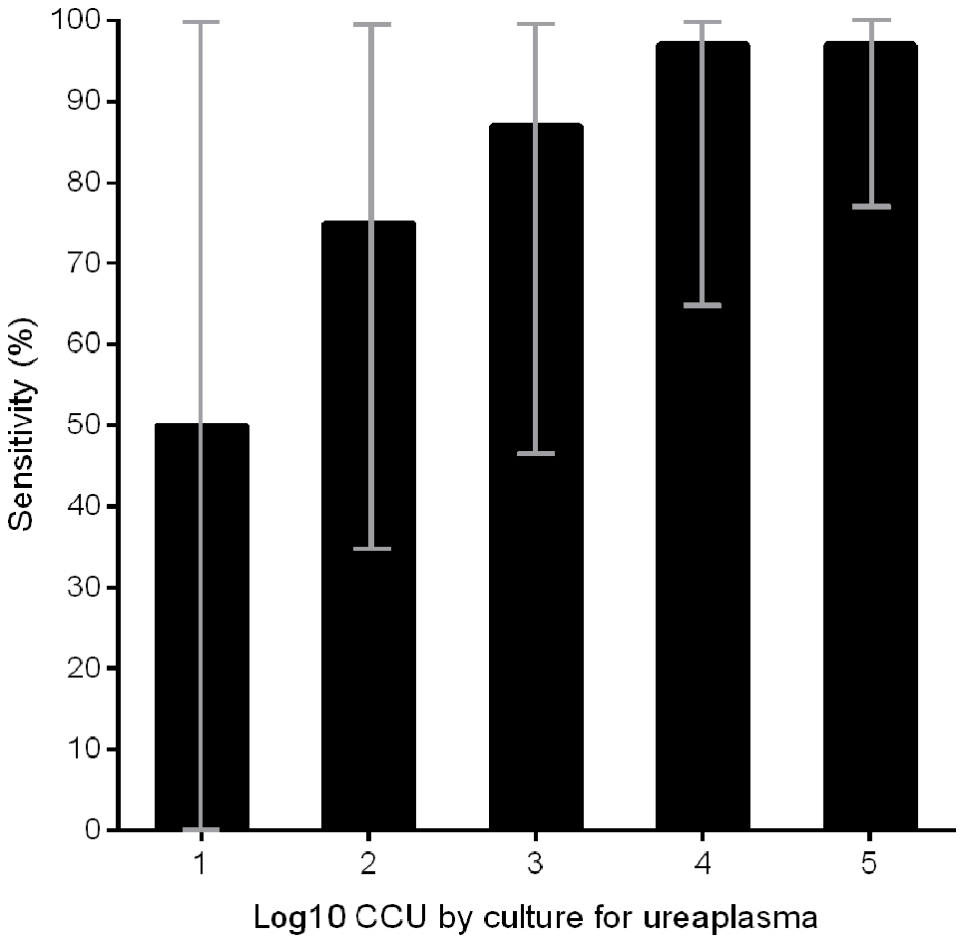
Bar-chart showing sensitivity in CCU group 1–5 for all sample types, male and female samples combined. CCU 1–5: 1 positive of 2 (50%), 9 of 12 (75%), 13 of 15 (87%), 28 of 29 (97%) and 70 of 72 (97%), respectively. Confidence intervals (95%) are depicted as grey vertical lines.

The overall sensitivity of the qPCR increased from 50% to 100% with increasing bacterial load determined by culture (Figure 2).

The predictive value of a positive qPCR test for both ureaplasma species combined was 98% (39 of 40) in male urethral swabs, 100% (43 of 43) in female urethral swabs and 95% (39 of 41) in cervical swabs. The corresponding negative predictive values were 94% (81 of 86), 89% (17 of 19) and 87% (13 of 15).

### Distribution of U. urealyticum and U. parvum

The distribution of the two ureaplasma species in qPCR and culture positive samples were 54% (21 of 39) *U. parvum* positives and 46% (18 of 39) *U. urealyticum* positives in male urethral swabs (Table 6). None of the men were infected with both *U. urealyticum* and *U. parvum*. In female urethral swabs the distribution was 70% (30 of 43) *U. parvum* positives and 23% (10 of 43) *U. urealyticum* positives, while 7% (3 of 43) were co-infected (Table 6). In the cervical swabs the distribution was 72% (28 of 39) *U. parvum* positives, 23% (9 of 39) *U. urealyticum* positives, while 5% (2 of 39) of the female urethral and cervical swabs were infected with both species, respectively (Table 6). Two female patients were co-infected in both urethral and cervical samples. There was no difference in the distribution of *U. parvum* positive or co-infected samples in the three sample types combined, while a trend towards more *U. urealyticum* positives in the male urethral samples was seen (p = 0.05) (Table 6). Forty-two female urethral and cervical swabs (pairs from the same patients) were positive by qPCR, and in all but one patient the same species was found in both specimens. The single non-concordant pair was co-infected in the urethral swab (1.2×10^3^ *U. urealyticum* DNA copies/µl and 2.4×10^2^ *U. parvum* DNA copies/µl), but only positive for *U. urealyticum* in the cervical swab. Three other pairs were co-infected, and species identification agreed for all.

**Table 6:** Distribution of *U. urealyticum* and *U. parvum* by qPCR in culture positive male and female urethral samples and cervical samples.

		No. of positives (%)		
	Total N	*U. parvum*	*U. urealyticum*	*U. parvum* and *U. urealyticum* *
Urethra (M)	39	21 (54)	18 (46)	0 (0)
Urethra (W)	43	30 (70)	10 (23)	3 (7)
Cervix (W)	39	28 (72)	9 (23)	2 (5)
All specimen types combined (%)	121	79 (65)	37 (31)	5 (4)
		p = 0.21	p = 0.05	p = 0.37

M =  man.

W =  woman.

* =  Infection with both *U. parvum* and *U. urealyticum*.

### Performance of the qPCR in Dual Infection

In the simulated samples with mixtures of known amounts of *U. urealyticum* and *U. parvum* DNA both species were detected in ratios up to 1∶100 and 100∶1 (*U. urealyticum: U. parvum*), using 100∶10,000 and 1,000∶100,000, and 1,000∶10 and 100,000∶1,000 DNA copies/µl. With the ratio 1,000∶1 with 1,000,000∶1,000 DNA copies/µl *U. parvum* was detected in 1 of 2 duplicates.

The five samples from women with co-infection had *U. urealyticum*:* U. parvum* ratios of 1∶5, 1∶38, 1∶2, 1∶30 and 7∶1 ranging from approximately 1 to 1.8×10^3^ DNA copies/µl.

### Comparison of quantitation by qPCR and culture

**Figure 3:**
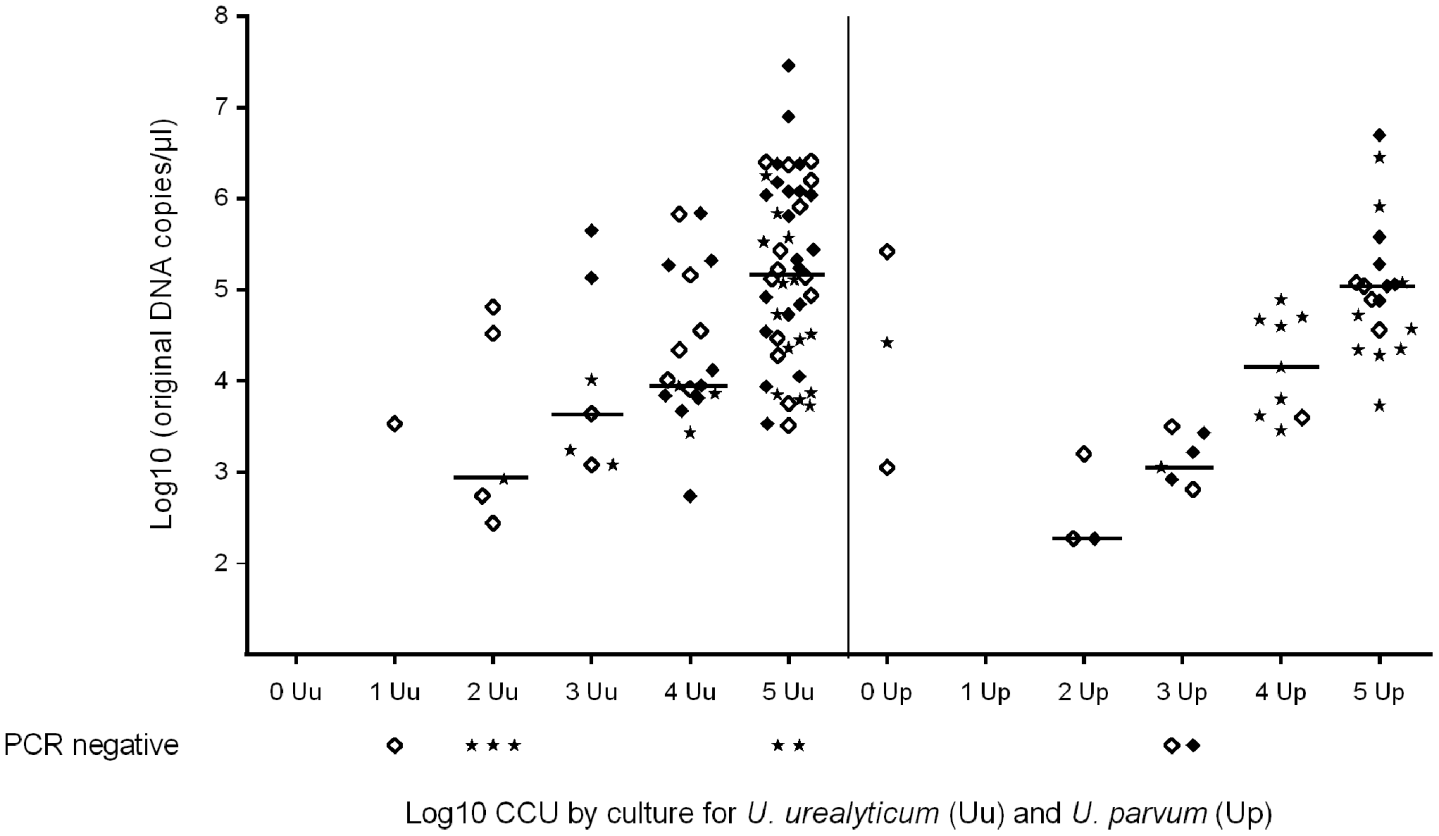
Comparison of *U. urealyticum* and *U. parvum* DNA load by quantitative real-time PCR and culture. ◊ =  female cervical swabs. ♦ =  female urethral swab. * =  male urethral swab. A total of 111 samples negative by both culture and qPCR, and one sample, which was culture positive in CCU group 4 but not re-detected with PCR, are not shown, as the species could not be determined. Co-infected specimens (5 samples) are not shown. Medians are depicted by a horizontal bar.

When the ureaplasma load, as determined by qPCR, was adjusted for differences in the inoculum size, the median calculated *U. urealyticum* DNA copy-number per 200 µl for all sample types combined would be approximately 8.8×10^2^, 4.3×10^3^, 9.5×10^3^ and 1.5×10^5^ for specimens containing 10^2^–10^5^ CCU (corresponding log10: 2.9, 3.6, 4.0 and 5.2), respectively. For *U. parvum* the corresponding medians would be 1.9×10^2^, 1.3×10^3^, 1.4×10^4^ and 1.1×10^5^ (log10: 2.3, 3.1, 4.2 and 5.0). The medians in 10^2^–10^5^ CCU did not differ between *U. urealyticum* and *U. parvum* when combining all sample types (p = 0.21, p = 0.07, p = 0.67 and p = 0.41, respectively). Medians are shown in Figure 3. When all samples were added, the median DNA load of *U. parvum* was 4.0×10^2^ DNA copies/µl and that of *U. urealyticum* 2.7×10^2^ DNA copies/µl (p = 0.045). The *U. parvum* median DNA load was higher in female urethral swabs than in male urethral swabs (1.3×10^3^ and 7.5×10^1^ DNA copies/µl, respectively [p = 0.004]), while the *U. urealyticum* DNA load in these groups did not differ (6.9×10^2^ and 2.2×10^2^ DNA copies/µl [p = 0.80]).

When sample types were combined, detection of *U. urealyticum* and *U. parvum* with qPCR, showed a nearly linear increasing median bacterial copy number with higher CCU (Figure 3). Linear regression showed no interaction between detection of *U. urealyticum* and *U. parvum* (p = 0.11).

### Analysis of Discrepancies between qPCR and Culture

After qPCR and culture results were obtained, 12 sample outcomes were discrepant (Table S1); Three samples were initially culture negative, but positive by qPCR; one was culture positive at repeat testing and all three were confirmed as true positives based on repeat positive qPCR and 16S rRNA gene PCR with the identical species in both swab and urine. Nine samples were culture positive but qPCR negative; seven were confirmed positive by repeat culture and six of these were qPCR positive from both swab and urine suggesting false negative qPCR in the initial run due to errors or low organism load. One specimen was negative in all tests at repeat sampling and most likely represents a sample mislabelling at the initial culture. One sample was lost, but the corresponding urine sample was positive by qPCR suggesting that the initial qPCR on the swab was false negative, probably due to an organism load below the limit of detection. Detailed results from re-testing are presented in table S1.

## Discussion

Ureaplasmas have been inconsistently associated with NGU and pregnancy complications [26]. Distinction between the two species of ureaplasmas is essential in a research setting and may also prove important in a clinical setting. Besides providing a quick result compared with culture, which takes up to 5 days, qPCR provides a quantitative estimate of the bacterial load in a sample, which is not possible by conventional PCR. Furthermore, conventional culture techniques cannot differentiate the two ureaplasma species and is sometimes invalidated by overgrowth of other bacteria.

The sensitivity of the qPCR was 96% and 95% in female urethral and cervical swabs, respectively. In male urethral swabs, the sensitivity was 89%. This is in very close agreement with previous studies comparing PCR and culture for detection of ureaplasmas. Blanchard *et al*. found an overall sensitivity of 92% [27], and Povlsen *et al.* found a sensitivity of 96% for undifferentiated female specimens and 91% for specimens from male patients [18]. In contrast to Povlsen *et al*. we found no difference in the sensitivity for the two ureaplasma species. In the present study mainly specimens with a low bacterial load as determined by culture were negative by qPCR. One of the main reasons for the lower sensitivity in such specimens resides in the inherent difficulties of PCR in accommodating a larger proportion of the clinical specimen. Whereas the sample preparation used in this study allowed an input volume of template corresponding to less than 2 µl of the original sample, the input volume for culture was more than 100-fold higher, as 200 µl of the original sample was used as inoculum. Thus, in theory, specimens with <100 CCU should not be detectable by PCR. However, as viability is not 100%, and as PCR may also detect non-viable ureaplasma cells, the sensitivity appears higher than theoretically possible. Obviously, alternative sample preparation methods that allow test of a larger volume of the original sample, could increase the sensitivity but at the cost of a possible higher risk of inhibition. Apart from speed, the PCR assay also has the advantage of being capable of analysing specimens, that are overgrown by other bacteria in culture, and thus, not evaluable by this technique. This was found to be the case in 5% overall in this study but in as many as 8% of the cervical swab specimens suggesting, that this specimen type may be particularly difficult to study by traditional culture. Of the 12 specimens overgrown by other bacteria 4 were positive by qPCR. If these are counted as true positive, the diagnostic yield of the qPCR would be 50% positives (128/256) and that of culture 51% (130/256).

The specificity was 99% for male urethral swabs and 100% for female urethral swabs, whereas it was 87% for cervical swab specimens. However, the number of negative specimens was small, particularly for female specimens, and in total only three qPCR positive/culture negative specimens were found. All three could be considered true positive based on findings with the 16S rRNA gene based PCR [17]. Since PCR also detects non-viable bacteria, the finding can be explained by the bacteria dying during transport from the clinic to the laboratory. The fact that the matching urethral swabs were both culture and PCR positive also suggests, that the initial cervical swab results were false negative by culture.

The qPCR results were comparable with the CCU groups for both species. However, the correlation appeared to be slightly better for *U. parvum*, as the qPCR ranges in 10^2^–10^5^ CCUs were wider for *U. urealyticum* than for *U. parvum*. Whether this reflects, that some strains of *U. urealyticum* are more fastidious, remains speculative, but since this species is more genetically heterogeneous [28],[29], it may be biologically plausible.

The quantitative aspect provided by the qPCR could be useful in clarifying the pathogenic potential of the two species in clinical samples. It has been shown, that repeated exposure to ureaplasmas leads to diminished urethritis signs and symptoms [30], and that *U. urealyticum* is associated with NGU in men with fewer lifetime sexual partners [31] suggesting, that symptoms may develop primarily upon initial exposure, and that it may be followed by an asymptomatic colonization in those failing spontaneous clearance or microbiological eradication after antibiotic treatment of the infection. A higher bacterial load of *U. urealyticum*, as compared to *U. parvum*, found in men with NGU would support these findings, and a stronger association with NGU would be expected in men with fewer lifetime sexual partners.

Our results from testing mixtures of *U. urealyticum* and *U. parvum* indicated, that the ureaplasma species with the lowest titer in a co-infected patient would not be detected, when the *U. urealyticum*: *U. parvum* ratios were more than either 1∶100 or 100∶1. However, we only found co-infections with ratios up to 1∶38, but whether that reflects inability to see larger differences in clinical specimens cannot be determined from the present study, as no attempt was made to detect the two species separately. Of the culture positive samples, 7% and 5% of the female urethral and cervical samples contained both species, respectively. Other studies have found ureaplasma co-infections in 8% (5/62) of female vaginal swabs and 4% (12/270) of male urine specimens with separate PCRs for the two ureaplasma species [31],[32]. This suggests, that the rate of co-infections, found in our study, reflects the true distribution.

In conclusion, the multiplex quantitative PCR provided rapid and accurate quantitation compared to culture and allowed detection of ureaplasmas also in specimens overgrown by other bacteria. Future studies should address the clinical relevance of the ureaplasma species and their quantitation when distinguishing between colonization and infection.

## Supporting Information

Table S1 **Result of re-testing 12 original samples with discrepant culture and qPCR results and the matching urine samples.** Results are given as colour-changing units (CCU) and as DNA copies/200 µl of the transport medium to adjust for differences in the amount of template or inoculum. (DOCX)

## References

[1] Shepard MC, Lunceford CD, Ford DK, Purcell RH, Taylor-Robinson D, et al (1974) *Ureaplasma urealyticum* gen. nov., sp. nov.: Proposed nomenclature for the human T (T-strain) Mycoplasmas. Int J Syst Bacteriol 24: 160–171.

[2] Robertson JA, Chen MH (1984) Effects of manganese on the growth and morphology of *Ureaplasma urealyticum* . J Clin Microbiol 19: 857–864. 6432835 10.1128/jcm.19.6.857-864.1984 PMC271199

[3] Kong FR, James C, Ma ZF, Gordon S, Bin W, Gilbert GL (1999) Phylogenetic analysis of Ureaplasma urealyticum - support for the establishment of a new species, Ureaplasma parvum. International Journal of Systematic Bacteriology 49: 1879–1889. 10555372 10.1099/00207713-49-4-1879

[4] Robertson JA, Stemke GW, Davis JW Jr, Harasawa R, Thirkell D, et al (2002) Proposal of *Ureaplasma parvum* sp. nov. and emended description of *Ureaplasma urealyticum* (Shepard, et al. 1974) Robertson, et al. 2001. Int J Syst Evol Microbiol 52: 587–597. 11931172 10.1099/00207713-52-2-587

[5] Wong JL, Hines PA, Brasher MD, Rogers GT, Smith RF, et al (1977) The etiology of nongonococcal urethritis in men attending a venereal disease clinic. Sex Transm Dis 4: 4–8. 867205 10.1097/00007435-197701000-00002

[6] Povlsen K, Bjornelius E, Lidbrink P, Lind I (2002) Relationship of *Ureaplasma urealyticum* biovar 2 to nongonococcal urethritis. Eur J Clin Microbiol Infect Dis 21: 97–101. 11939406 10.1007/s10096-001-0665-1

[7] Ondondo RO, Whittington WL, Astete SG, Totten PA (2010) Differential association of ureaplasma species with non-gonococcal urethritis in heterosexual men. Sex Transm Infect 86: 271–275 sti.2009.040394 [pii]; 10.1136/sti.2009.040394 [doi] 20460265 PMC4628822

[8] Yamazaki T, Matsumoto M, Matsuo J, Abe K, Minami K, et al. (2012) Frequency of Chlamydia trachomatis in Ureaplasma-positive healthy women attending their first prenatal visit in a community hospital in Sapporo, Japan. Bmc Infectious Diseases 12.. 10.1186/1471-2334-12-82 PMC3342208 22471518

[9] Bowie WR, Wang SP, Alexander ER, Floyd J, Forsyth PS, et al (1977) Etiology of nongonococcal urethritis. Evidence for *Chlamydia trachomatis* and *Ureaplasma urealyticum* . J Clin Invest 59: 735–742. 300742 10.1172/JCI108694 PMC372280

[10] Horner P, Thomas B, Gilroy CB, Egger M, Taylor-Robinson D (2001) Role of *Mycoplasma genitalium* and *Ureaplasma urealyticum* in acute and chronic nongonococcal urethritis. Clin Infect Dis 32: 995–1003. 11264026 10.1086/319594

[11] Kafetzis DA, Skevaki CL, Skouteri V, Gavrili S, Peppa K, et al (2004) Maternal genital colonization with Ureaplasma urealyticum promotes preterm delivery: association of the respiratory colonization of premature infants with chronic lung disease and increased mortality. Clin Infect Dis 39: 1113–1122 CID32584 [pii]; 10.1086/424505 [doi] 15486833

[12] Kasper DC, Mechtler TP, Bohm J, Petricevic L, Gleiss A, et al (2011) In utero exposure to Ureaplasma spp. is associated with increased rate of bronchopulmonary dysplasia and intraventricular hemorrhage in preterm infants. Journal of Perinatal Medicine 39: 331–336. 21526978 10.1515/jpm.2011.022

[13] Deguchi T, Yoshida T, Miyazawa T, Yasuda M, Tamaki M, et al (2004) Association of *Ureaplasma urealyticum* (biovar 2) with nongonococcal urethritis. Sex Transm Dis 31: 192–195. 15076934 10.1097/01.olq.0000114653.26951.71

[14] Shimada Y, Ito S, Mizutani K, Sugawara T, Seike K, et al.. (2013) Bacterial loads of Ureaplasma urealyticum contribute to development of urethritis in men. Int J STD AIDS. 0956462413504556 [pii];10.1177/0956462413504556 [doi]. 10.1177/0956462413504556 24047884

[15] Bradshaw CS, Tabrizi SN, Read TR, Garland SM, Hopkins CA, et al (2006) Etiologies of nongonococcal urethritis: bacteria, viruses, and the association with orogenital exposure. J Infect Dis 193: 336–345. 16388480 10.1086/499434

[16] Carne CA, Gibbs J, Delaney A, Sonnex C, Verlander NQ, et al (2013) Prevalence, clinical features and quantification of genital non-viral infections. Int J STD AIDS 24: 273–277 24/4/273 [pii]; 10.1177/0956462412472306 [doi] 23970658

[17] Robertson JA, Vekris A, Bebear C, Stemke GW (1993) Polymerase chain reaction using 16S rRNA gene sequences distinguishes the two biovars of *Ureaplasma urealyticum* . J Clin Microbiol 31: 824–830. 7681846 10.1128/jcm.31.4.824-830.1993 PMC263571

[18] Povlsen K, Jensen JS, Lind I (1998) Detection of *Ureaplasma urealyticum* by PCR and biovar determination by liquid hybridization. J Clin Microbiol 36: 3211–3216. 9774567 10.1128/jcm.36.11.3211-3216.1998 PMC105303

[19] Xiao L, Glass JI, Paralanov V, Yooseph S, Cassell GH, et al (2010) Detection and characterization of human Ureaplasma species and serovars by real-time PCR. J Clin Microbiol 48: 2715–2723 JCM.01877-09 [pii]; 10.1128/JCM.01877-09 [doi] 20554828 PMC2916572

[20] Yoshida T, Deguchi T, Meda S, Kubota Y, Tamaki M, et al (2007) Quantitative detection of Ureaplasma parvum (biovar 1) and Ureaplasma urealyticum (biovar 2) in urine specimens from men with and without urethritis by real-time polymerase chain reaction. Sex Transm Dis 34: 416–419. 17522569 10.1097/01.olq.0000243621.89212.40

[21] Jensen JS, Björnelius E, Dohn B, Lidbrink P (2004) Comparison of first void urine and urogenital swab specimens for detection of *Mycoplasma genitalium* and *Chlamydia trachomatis* by polymerase chain reaction in patients attending a sexually transmitted disease clinic. Sex Transm Dis 31: 499–507. 15273584 10.1097/01.olq.0000135992.98883.e4

[22] Tully JG, Whitcomb RF, Clark HF, Williamson DL (1977) Pathogenic mycoplasmas: cultivation and vertebrate pathogenicity of a new spiroplasma. Science 195: 892–894. 841314 10.1126/science.841314

[23] Shepard MC (1983) Culture media for ureaplasmas. In: Razin S, Tully JG, editors. Methods in Mycoplasmology. New York: Academic Press, Inc. pp. 137–146.

[24] Kong F, James G, Ma Z, Gordon S, Bin W, et al (1999) Phylogenetic analysis of Ureaplasma urealyticum–support for the establishment of a new species, Ureaplasma parvum. Int J Syst Bacteriol 49 Pt 41879-89: 89. 10.1099/00207713-49-4-1879 10555372

[25] Jensen JS, Björnelius E, Dohn B, Lidbrink P (2004) Use of TaqMan 5′ nuclease real-time PCR for quantitative detection of *Mycoplasma genitalium* DNA in males with and without urethritis who were attendees at a sexually transmitted disease clinic. J Clin Microbiol 42: 683–692. 14766837 10.1128/JCM.42.2.683-692.2004 PMC344445

[26] Totten PA, Taylor-Robinson D, Jensen JS (2008) Genital mycoplasmas. In: Holmes KK, Sparling PF, Stamm WE, Piot P, Wasserheit JN, et al.., editors. Sexually Transmitted Diseases. New York: McGraw Hill. pp. 709–736.

[27] Blanchard A, Hentschel J, Duffy L, Baldus K, Cassell GH (1993) Detection of *Ureaplasma urealyticum* by Polymerase Chain Reaction in the Urogenital Tract of Adults, in Amniotic Fluid, and in the Respiratory Tract of Newborns. Clin Infect Dis 17: S148–S153. 8399906 10.1093/clinids/17.supplement_1.s148

[28] Robertson JA, Pyle LE, Stemke GW, Finch LR (1990) Human ureaplasmas show diverse genome sizes by pulsed-field electrophoresis. Nucleic Acids Res 18: 1451–1455. 2326188 10.1093/nar/18.6.1451 PMC330511

[29] Paralanov V, Lu J, Duffy LB, Crabb DM, Shrivastava S, et al (2012) Comparative genome analysis of 19 Ureaplasma urealyticum and Ureaplasma parvum strains. BMC Microbiol 12: 88 1471-2180-12-88 [pii]; 10.1186/1471-2180-12-88 [doi] 22646228 PMC3511179

[30] Taylor-Robinson D (1996) The history of nongonococcal urethritis. Thomas Parran Award Lecture. Sex Transm Dis 23: 86–91. 8801649 10.1097/00007435-199601000-00020

[31] Wetmore CM, Manhart LE, Lowens MS, Golden MR, Jensen NL, et al (2011) *Ureaplasma urealyticum* is associated with nongonococcal urethritis among men with fewer lifetime sexual partners: a case-control study. J Infect Dis 204: 1274–1282 jir517 [pii]; 10.1093/infdis/jir517 [doi] 21917901 PMC3173507

[32] Ekiel AM, Friedek DA, Romanik MK, Jozwiak J, Martirosian G (2009) Occurrence of Ureaplasma parvum and Ureaplasma urealyticum in Women with Cervical Dysplasia in Katowice, Poland. Journal of Korean Medical Science 24: 1177–1181. 19949678 10.3346/jkms.2009.24.6.1177 PMC2775870

