# Supplementary material for: Comparison between Culture and a Multiplex Quantitative Real-Time Polymerase Chain Reaction Assay Detecting Ureaplasma urealyticum and U. parvum
Source: PLoS One. 2014 Jul 21;9(7):e102743. doi: 10.1371/journal.pone.0102743 (PMC4105565; doi:10.1371/journal.pone.0102743)
Supplement: Table S1 — (DOCX) [file pone.0102743.s001.docx]

| Sample number | Sample type | Result  (CCU / qPCR swab) | 16S rRNA gene PCR (swab) | Re-testing  (CCU / qPCR swab / qPCR urine) | 16S rRNA gene PCR (swab / urine) | Possible explanation for discrepancy |
| --- | --- | --- | --- | --- | --- | --- |
| 1 | Male urethral swab | - / ~4x10^4^ | + | 10^5^ / ~8x10^3^ / ~10^3^ | + / - | Error during culture |
| 2 | Cervical swab^a^ | - / ~4x10^5^ | - | - / ~10^3^ / ~9x10^3^ | - / + | Non-viable ureaplasmas |
| 3 | Cervical swab^a^ | - / ~2x10^3^ | - | - / - / ~10^2^ | - / + | Non-viable ureaplasmas |
| 4 | Male urethral swab | 10^2^ / - | - | 10^3^ / - / ~10^1^ | - / - | Below limit of detection for PCR |
| 5 | Male urethral swab | 10^2^ / - | - | 10^2c^ / - / - | - / - | Below limit of detection for PCR |
| 6 | Male urethral swab | 10^2^ / - | - | ND^b^ / ND^b^ / ~10^1^ | ND^b^ / - | Below limit of detection for PCR |
| 7 | Male urethral swab | 10^5^ / - | - | 10^5^ / ~8x10^3^ / ~2x10^5^ | + / + | Error during qPCR |
| 8 | Male urethral swab | 10^5^ / - | - | 10^5^ / ~10^5^ / ~4x10^3^ | + / + | Error during qPCR |
| 9* | Female urethral swab | 10^3^ / - | - | 10^3^ / ~10^3^ / ~10^4^ | + / + | Close to limit of detection for PCR |
| 10* | Cervical swab | 10^3^ / - | - | 10^3^ / ~10^2^ / ~10^4^ | + / + | Close to limit of detection for PCR |
| 11 | Cervical swab^a^ | 10^1^ / - | + | 10^2c^/ - / ~4x10^3^ | - / + | Below limit of detection for PCR |
| 12 | Female urethral swab | 10^4^ / - | - | - / - / - | - / - | Error during culture |

**Table S1.** Result of re-testing 12 original samples with discrepant culture and qPCR results and the matching urine samples. Results are given as colour-changing units (CCU) and as DNA copies/200μl of the transport medium to adjust for differences in the amount of template or inoculum.
